# Enhancing Char Formation and Flame Retardancy of Ethylene-Vinyl Acetate Copolymer (EVA)/Aluminum Hydroxide (ATH) Composites by Grafting Ladder Phenyl/Vinyl Polysilsesquioxane (PhVPOSS)

**DOI:** 10.3390/polym15153312

**Published:** 2023-08-05

**Authors:** Fa Hu, Bo Cheng, Kun Cong, Dinghua Li, Wenchao Zhang, Zhaolu Qin, Rongjie Yang

**Affiliations:** 1 National Engineering Research Center of Flame Retardant Materials, School of Materials Science and Engineering, Beijing Institute of Technology, Beijing 100081, China; huf.bjhy@sinopec.com (F.H.);; 2 SINOPEC (Beijing) Research Institute of Chemical Industry Co., Ltd., Beijing 100013, China; 3 China Petroleum Engineering & Construction Corporation, Beijing 100120, China

**Keywords:** reactivity, char formation, flame retardancy, smoke suppression, EVA

## Abstract

The ladder phenyl/vinyl polysilsesquioxane (PhVPOSS) was used to improve the flame-retardancy performances of ethylene-vinyl acetate copolymer (EVA)/aluminum hydroxide (ATH) composites due to the reactivity of its vinyl groups. FTIR, XPS, ^1^H NMR, and SEM-EDS data demonstrated the PhVPOSS grafting onto EVA molecular chains. The PhVPOSS improved the thermal stability of EVA/ATH composites, as shown by the thermogravimetric analysis (TGA). Furthermore, with the cone calorimeter (CONE) experiments, EVA/ATH/PhVPOSS showed better fire safety than the EVA/ATH composites, with the PHRR, PSPR, and PCOP reduced by 7.89%, 57.4%, and 90.9%, respectively. The mechanism investigations of flame retardancy revealed that the charring behaviors of the EVA/ATH/PhVPOSS composites were improved by the formation of Si-C bonds and Si-O bonds, and a more compact and denser char layer can contribute more to the barrier effect.

## 1. Introduction

Ethylene-vinyl acetate copolymer (EVA) is used in many fields, especially in the cable industry as an excellent insulating material, owing to its good physical and mechanical properties [1]. However, like most other organic materials, EVA is also combustible, thus limiting its application. Thus, it is necessary to improve the flame retardancy of EVA. Aluminum hydroxide (ATH) is the most widely used flame retardant due to its smoke suppression, non-toxicity, and low price [2,3]. Unfortunately, ATH usually exhibits low flame-retardant efficiency; thus, high loading is needed for decent flame retardancy, which commonly leads to the deterioration of the mechanical properties of the final products [4].

To solve the abovementioned problem, several researchers have attempted to partially replace ATH with other flame retardants to improve the flame-retardant efficiency and reduce the amount of required flame retardants [5,6,7,8,9,10,11,12,13,14,15]. Zhuo et al. [5] prepared silicone rubber/ATH composites with red phosphorus as a flame retardant; they found that a high content of red phosphorus led to significant improvements in flame retardancy through the limiting oxygen index (LOI), UL-94, and cone calorimetric experiments. Fang et al. [9] prepared polyethylene/ATH composites with fullerene (C_60_) as a flame retardant; the addition of a given amount of C_60_ increased the LOI value and UL-94 rating while greatly decreasing the heat release rate (HRR) and prolonging the combustion time. Although red phosphorus and fullerenes can improve the flame-retardant efficiency of ATH, red phosphorus tends to produce toxic gases during processing [16], and fullerenes can reduce the insulation of the polymer.

Polyhedral oligomeric silsesquioxanes (POSSs) are organic and inorganic hybrid materials with both organic functional groups and inorganic Si-O-Si structures, which usually have good compatibility with polymers and excellent thermal stability [17,18,19]. As efficient synergist agents, POSSs can significantly improve flame retardant efficiency without producing harmful gases during processing and reducing the insulation properties of the polymer composites [20,21,22,23,24,25,26,27,28]. Feng et al. [27] partially replaced aluminum diethylphosphonate (ADP) by using octaphenyl polyhedral oligomeric silsesquioxanes (OPS) in PA6, and the peak heat release rate (PHRR) decreased by 60.2%, and the maximum smoke density (Ds max) decreased by 45.9%, when the total addition reached 10%. Didane et al. [29] applied a 9:1 ratio of ADP and octamethyl POSS (OMPOSS) to PET flame retardant, and the PHRR decreased by 49% when the addition was 10%. Therefore, the combination of POSS and ATH is considered to improve the flame retardancy of the materials.

In addition, POSSs can be designed with various organic functional groups to generate varied properties. Vinyl-containing POSSs are reactive and can be grafted onto polymer chain segments during polymer processing. Fina et al. [30] physically blended vinyl POSSs with polypropylene (PP) and then removed the free vinyl POSSs. The presence of a characteristic peak of POSS in the infrared spectrum of PP proved that vinyl POSSs were grafted onto the PP chain segment during processing. PP with vinyl POSSs has improved mechanical properties, thermal stability, and flame retardancy. Similarly, the different POSS structures also lead to different properties. Zhang et al. [31] demonstrated that ladder phenyl POSSs had greater thermal stability than cage POSSs, as the residue mass of ladder phenyl POSSs was 5.4 times more than that of cage phenyl POSSs at 800 °C. Based on the above analysis, ladder POSSs with vinyl may further improve the performance of flame-retardant materials.

In our laboratory, the ladder vinyl/phenyl POSS was synthesized [21]. Its polymerization degree was 6 or 11, and the vinyl/phenyl ratio was 1:2 or 2:9. Herein, this ladder phenyl/vinyl polysilsesquioxane (PhVPOSS) was introduced to EVA/ATH composites. The grafting of PhVPOSS onto the EVA segment was demonstrated by various characterization methods. PhVPOSS significantly improved the thermal stability and flame retardancy of EVA composites. In particular, PhVPOSS significantly improved the charring behaviors of non-charring polyolefins during combustion. Finally, we proposed the detailed flame-retardant mechanism from the perspective of the condensed and gaseous phases.

## 2. Materials and Methods

### 2.1. Materials

**Figure 1:**
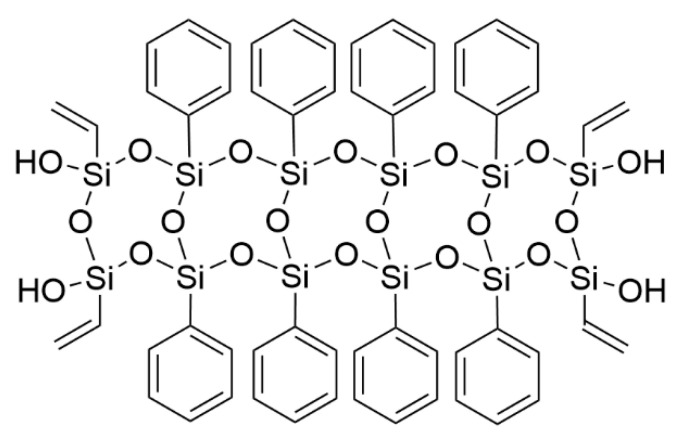
Chemical structure of polyphenyl/vinyl silsesquioxane (PhVPOSS).

The ethylene-vinyl acetate copolymer (EVA) used was the Bycolene V5110J grade from BASF-YPC Company Limited (Nanjing, China), with a melt flow index of 2.3–3.1 g/10 min (190 °C and 2.16 kg) and a vinyl acetate content of 18.5 wt.%. Aluminum hydroxide (ATH) AITEMAG A140FD ground-milled grade was supplied by Jiangsu ATK Flame Retardant Material Co. Ltd. (Wuxi, China), with an average diameter of 1.8 μm. PhVPOSSs were prepared from BITFR Co., Ltd. (Beijng, China), according to our previous works [21]. The chemical structure of PhVPOSS is shown in Figure 1. All materials were used as received, without further purification.

### 2.2. Preparation of the EVA/ATH/PhVPOSS Composites

All EVA composites were prepared in a EHT 50 twin-roller mill (Brabender GmbH & Co., KG., Duisburg, Germany) at 170 °C for 10 min. After mixing, the samples were hot pressed at 110 °C under 10 MPa for 5 min into sheets of suitable thickness for further characterization. The formulations of the composites are given in Table 1.

### 2.3. Characterization and Measurement

The infrared spectra of the samples were obtained at ambient temperature, using Fourier-transform infrared spectroscopy (FTIR, ThermoFisher SCIENTIFIC, Waltham, MA, USA). Single-beam spectra of the samples were obtained after an average of 32 scans between 4000 and 400 cm^−1^. All spectra were obtained in the transmittance mode. The ^1^H NMR spectra were obtained from a Bruker Advance 600 NMR spectrometer. CDCl_3_ was used as the solvent for PhVPOSS and EVA composites. The dispersion state of PhVPOSS in the EVA was characterized by a Hitachi S-4800 scanning electron microscope (SEM) with energy-dispersive spectroscopy (EDS) in high vacuum mode with a 15 kV acceleration voltage. 

Thermogravimetric analysis (TGA) was carried out using a TGA-Q500 thermal analyzer (TA, New Castle, DE, USA) in nitrogen from 40 to 800 °C with a heating rate of 10 °C/min. A 5 mg sample was used for each measurement. The initial decomposition temperature is defined from the 5% mass loss (T_5_%); R_max_ and T_max_, which are the maximum decomposition rate and temperature at that point, were obtained. T_5_% and the solid residue are obtained from TGA curves; R_max_ and T_max_ were obtained from DTG curves.

The limiting oxygen index (LOI) was tested using an oxygen index instrument (Rheometric Scientific Ltd., Hampshire, UK) with dimensions of 100 × 6.5 × 3 mm^3^ according to ASTM D 2863-08 standard. The vertical burning test was conducted using a CZF-3 horizontal and vertical burning tester (Jiang Ning Analysis Instrument Company, Nanjing, China) on sheets 125 × 12.5 × 3 mm^3^ according to UL-94 test ASTM D3801. Cone calorimeter (CONE) measurements were performed under 35 kW/m^2^ heat flux, using an FTT cone calorimeter (Fire Testing Technology, East Grinstead, UK), according to ISO 5660, with sheet dimensions of 100 × 100 × 3 mm^3^. All tests were performed at least three times to check reproducibility, and then the heat release rate (HRR), total heat release (THR), smoke production rate (SPR), total smoke release (TSR), time to ignition (TTI), maximum HRR (PHRR), and maximum SPR (PSPR) were recorded simultaneously.

X-ray photoelectron spectroscopy (XPS) analyses were performed with a PHI 5300C ESCA System photoelectron spectrometer (PHI Corp), equipped with an Al Kα radiation source, at 250 W under a vacuum as high as 10^−6^ Pa (10^−8^ Torr) (Perkin-Elmer Corp, Waltham, MA, USA). Raman spectra of the char residue samples were recorded with an excitation line from a 514 nm source at room temperature. A thermogravimetry–Fourier-transform infrared spectrometer (TG-FTIR) test was performed with a 209 F1 thermal analyzer (TGA, Mettler Toledo TGA, Zurich, Switzerland) coupled with a Fourier-transform infrared spectrometer (FTIR, Thermo Scientific Nicolet iS50, Waltham, MA, USA). Samples were heated from 40 to 800 °C at a heating rate of 20 °C/min.

## 3. Results and Discussion

### 3.1. Grafting Reaction of PhVPOSS and EVA

**Figure 2:**
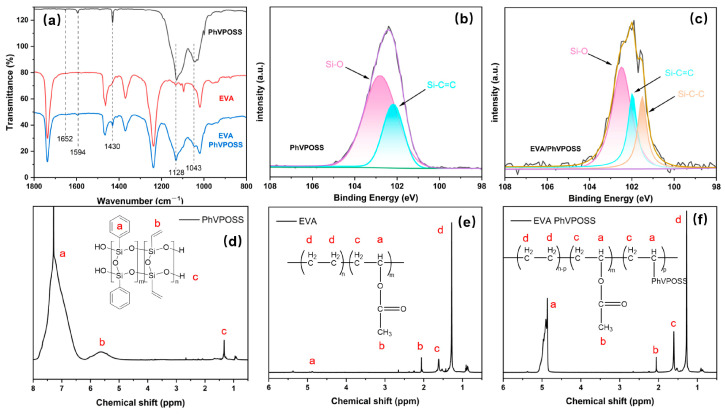
(**a**) FTIR spectra of the PhVPOSS, EVA, and EVA/PhVPOSS composite. High-resolution Si 2p XPS spectra of (**b**) PhVPOSS and (**c**) EVA/PhVPOSS composite. ^1^H NMR spectra of (**d**) PhVPOSS, (**e**) EVA, and (**f**) EVA/PhVPOSS composite.

The grafting reactivity during the blending processing of PhVPOSS and EVA was investigated by FTIR, XPS, and H-NMR, and the spectra are shown in Figure 2a–f. In Figure 2a, the weak absorption peaks at 1652 cm^−1^ and 1594–1430 cm^−1^ correspond to the stretching vibrations of the C=C bond on the vinyl and phenyl groups, and the absorption peaks at 1128 and 1043 cm^−1^ represent the Si-O-Si structure in the horizontal and vertical directions, respectively [32,33]. For the EVA/PhVPOSS composite, the stretching vibration peak of the vinyl disappeared at 1652 cm^−1^, indicating a reaction between PhVPOSS and EVA during high-temperature processing. Meanwhile, the appearance of Si-C-C bonds in the XPS spectra of the EVA/PhVPOSS composites demonstrated that PhVPOSS reacted during processing.

**Figure 3:**
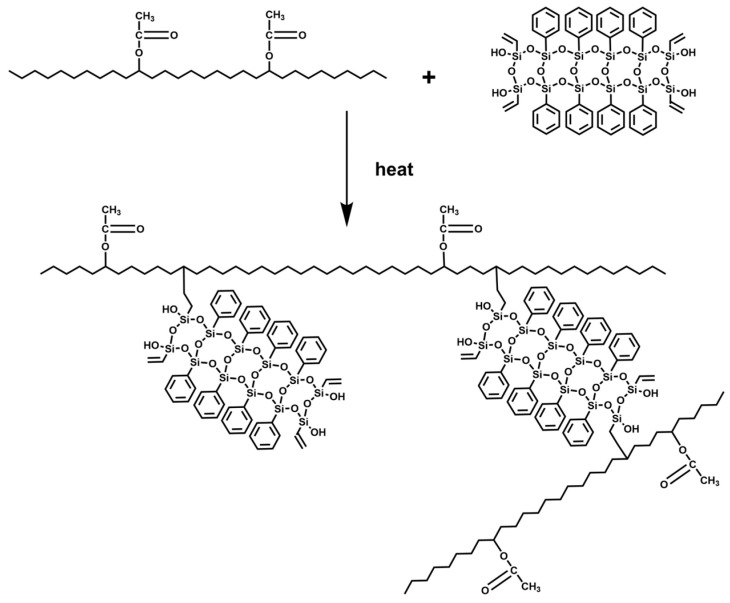
Reaction scheme of PhVPOSS grafting onto EVA.

The ^1^H NMR spectra were utilized to further investigate the reaction processes of PhVPOSS and EVA. In the EVA/PhVPOSS composite (Figure 2f), the peaks at 5.1–4.7 ppm were significantly enhanced. This was different from the characteristic peak on PhVPOSS (Figure 2d), so it is the hydrogen in the EVA chain segment after grafting EVA by PhVPOSS. Since the methylene peak was significantly weakened, PhVPOSS may have been grafted on the ethylene chain segment. That is, PhVPOSS was grafted onto the ethylene chain segment of EVA during processing (Figure 3).

**Figure 4:**
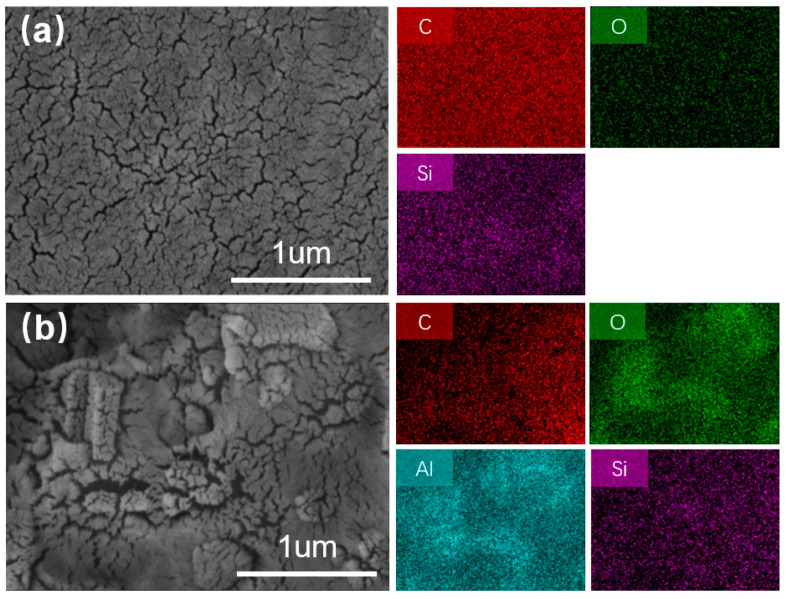
SEM images and the corresponding C, O, Al, and Si elemental mapping of the fracture surface of (**a**) EVA/PhVPOSS composites and (**b**) EVA/ATH/PhVPOSS composites.

The dispersion of the compound particles in the polymer correlates with the occurrence of grafting reactions. When grafting occurs, the aggregated particles break up into smaller particles for more uniform dispersion [32]. As shown in Figure 4a,b, PhVPOSS achieved micron-sized uniform dispersion in both the EVA and EVA/ATH composites. That is, a reaction occurred between PhVPOSS and EVA, and therefore a more homogeneous dispersion was produced.

### 3.2. Thermal Behavior of EVA/ATH/PhVPOSS

**Figure 5:**
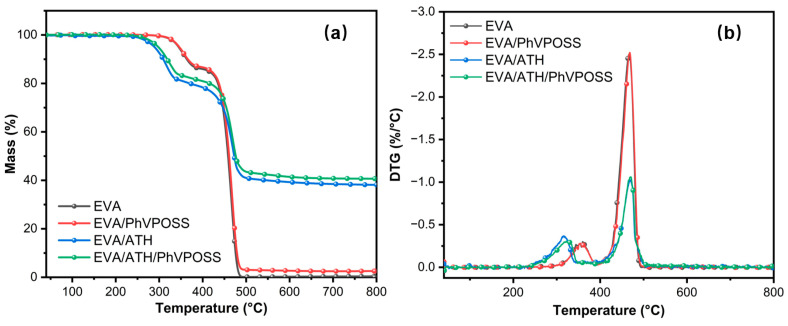
(**a**) TGA and (**b**) DTG curves of EVA, EVA/PhVPOSS, EVA/ATH, and EVA/ATH/PhVPOSS composites.

To investigate the influences of PhVPOSS on the thermostability of EVA and EVA/ATH composite, TGA under a N_2_ atmosphere was performed, and the results are shown in Figure 5 and Table 2. Pure EVA decomposition was divided into two stages, the removal of acetic acid groups between 300 and 400 °C and the breaking of polymer chain segments between 400 and 500 °C [34,35,36]. For EVA grafted with PhVPOSS, we observed a higher temperature value both for initial thermal decomposition and the maximum rate of mass loss, with a 2.16% increase in residue mass at 800 °C.

In EVA/ATH composites, the initial decomposition temperature of the EVA/ATH composite was significantly reduced because ATH begins to decompose at 253 °C [37]. The ATH decomposition product aluminum oxide has good thermal stability at high temperatures, and the residual char rate increased significantly at 800 °C. With the existence of PhVPOSS in EVA/ATH composites, the initial decomposition temperature, maximum rate of mass loss, and residual mass increased significantly, as well. That is, grafted PhVPOSS improves the thermal stability of EVA and the EVA/ATH composite.

### 3.3. Fire Behaviors of EVA/ATH/PhVPOSS Composites

The flammability of EVA composites was evaluated by UL-94 vertical burning tests and limiting oxygen index (LOI) tests. The data are summarized in Table 3. As for the UL-94 vertical burning rating, when PhVPOSS and ATH were added into EVA separately, both specimens were classified as having no rating (NR). However, EVA/ATH/PhVPOSS can achieve V-1 level. As for the LOI value, PhVPOSS does not show significant effect for both EVA and EVA/ATH. It indicates that the flame-retardance mechanism of PhVPOSS is likely mainly to function in the condensed phase, rather than in the gas phase.

**Figure 6:**
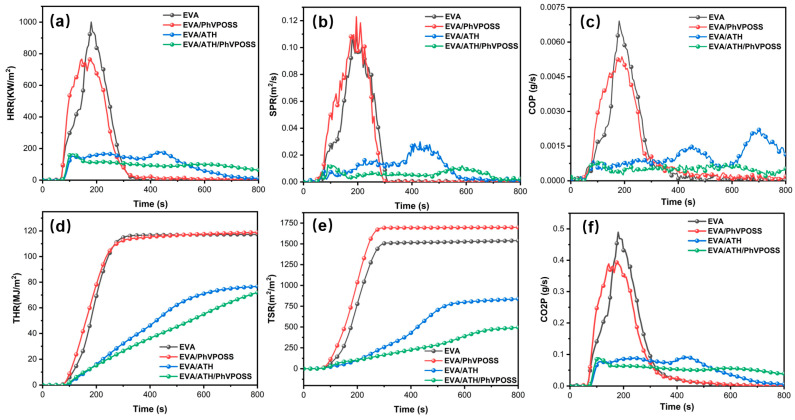
Cone curves of EVA, EVA/PhVPOSS, EVA/ATH, and EVA/ATH/PhVPOSS composites: (**a**) HRR, (**b**) SPR, (**c**) COP, (**d**) THR, (**e**) TSR, and (**f**) CO_2_P.

The CONE tests were carried out to investigate the emission of heat, smoke, and toxic gas (CO) during the combustion process of EVA, EVA/PhVPOSS, EVA/ATH, and EVA/ATH/PhVPOSS composites. As shown in Figure 6a and Table 4, compared with EVA, the PHRR values of EVA/PhVPOSS, EVA/ATH, and EVA/ATH/PhVPOSS composites were reduced by 23.4%, 81.9%, and 83.3%, respectively. Furthermore, as for the total heat release (THR), the introduction of PhVPOSS into EVA/ATH composites led to a significantly inhibited heat release cumulative value.

In a fire, smoke can impair people’s vision to the detriment of escape, and toxic gases can cause poisoning and suffocation. Therefore, the reduction of toxic smoke is of great importance in improving fire safety. SPR, TSR, and COP can reflect the toxic flue gas content. As shown in Figure 6b,c,e, compared with the EVA, when ATH and PhVPOSS were added together to EVA, the SPR, TSR, and COP decreased by 88.7%, 67.8%, and 97.1%, respectively. The above results show that the grafted PhVPOSS in EVA/ATH composites significantly enhance the fire safety of EVA/ATH composites.

### 3.4. Flame-Retardant Mechanism

#### 3.4.1. Char Residue Analysis

**Figure 7:**
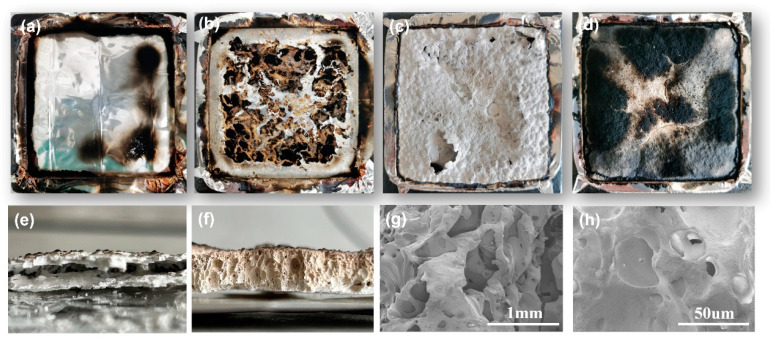
Digital images of char after CONE test for EVA (**a**), EVA/PhVPOSS (**b**), EVA/ATH (**c**), and EVA/ATH/PhVPOSS; (**d**) cross-sectional photos and SEM images of EVA/ATH (**e**,**g**) and EVA/ATH/PhVPOSS (**f**,**h**) composite residues.

The digital photographs and SEM images of the char residuals after the CONE tests are shown in Figure 7a–d. Pure EVA burned without any residue and exhibited the typical characteristics of a non-charring polymer. For the EVA grafted with PhVPOSS, the residue char was yellow and black. Similarly, when only ATH was added to EVA, only a large amount of loose white alumina powder was present after combustion; after the addition of PhVPOSS, yellow and black charring appeared on the surface. Figure 7e,f show cross-sectional photographs of the EVA/ATH and EVA/ATH/PhVPOSS composite residues, respectively; the EVA/ATH residue had a loose lamellar structure, while the EVA/ATH/PhVPOSS char residue was a more complete mass. That is, PhVPOSS enhanced the charring behaviors of EVA and EVA/ATH composites.

**Figure 8:**
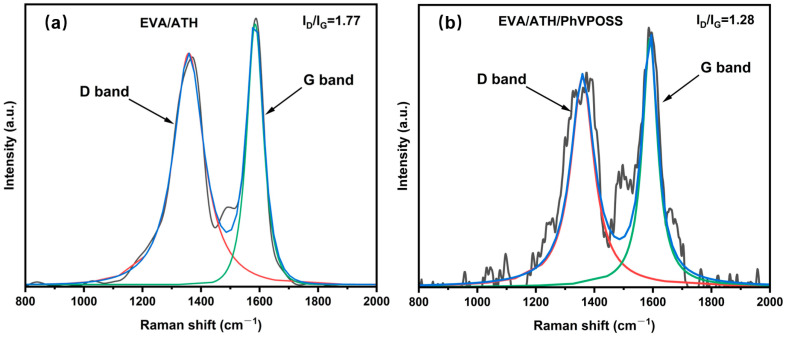
Raman spectra of char after CONE test for (**a**) EVA/ATH and (**b**) EVA/ATH/PhVPOSS.

Similarly, the char layer of the EVA/ATH/PhVPOSS composite was observed to be denser and less porous than EVA/ATH, as shown in SEM. Raman spectroscopy is often used to characterize the graphitization degree of the residual char; the lower the I_D_/I_G_, the higher the degree of graphitization and the denser the residual char [38,39,40,41]. As shown in Figure 8, the ID/IG value for EVA/ATH/PhVPOSS decreased from 1.77 to 1.28 compared to EVA/ATH composites. The above results demonstrated that the EVA/ATH/PhVPOSS composite has a denser char layer and a better barrier effect.

**Figure 9:**
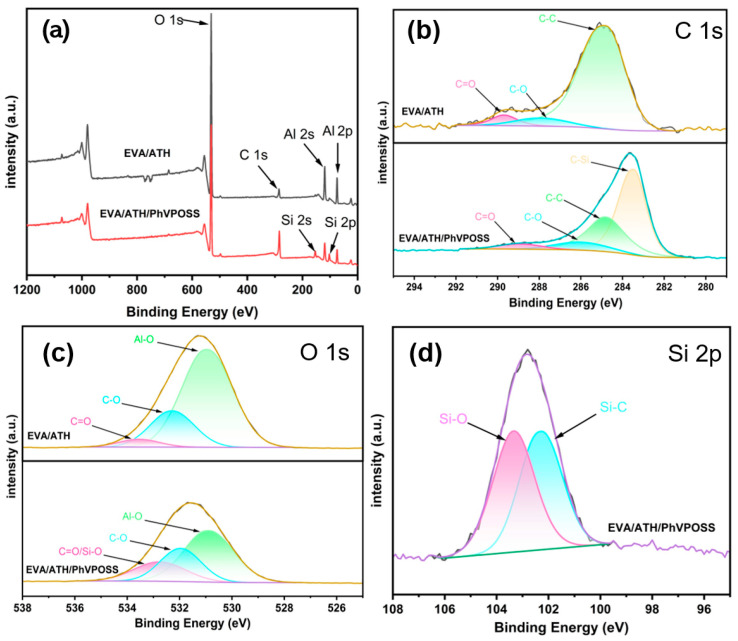
(**a**) XPS survey spectra and (**b**) C 1s, (**c**) O 1s, and (**d**) Si 2p XPS spectra of chars for EVA/ATH and EVA/ATH/PhVPOSS composites.

The chemical composition and the oxidation states of char elements in the EVA/ATH and EVA/ATH/PhVPOSS were determined by XPS, and the spectra are shown in Figure 9. The full scan survey spectra (Figure 9a) show only O 1s, C 1s, Al 2s, and Al 2p peaks in the EVA/ATH char and additional Si 2s and Si 2p peaks in the EVA/ATH/PhVPOSS char. The narrow scanning C 1s, O 1s, and Si 2p spectra of residual chars are shown in Figure 9b–d. Compared to EVA/ATH, the C spectrum of EVA/ATH/PhVPOSS residual char showed four peaks at 288.92 ev, 285.97 ev, 284.8 ev, and 283.46 ev, and these correspond to the C=O, C-O, C-C/C=C, and C-Si groups, respectively. Compared to EVA/ATH, the O spectrum of EVA/ATH/PhVPOSS residual char showed three peaks at 530.88 ev, 531.96 ev, and 532.76 ev, and these correspond to the Al-O, C-O, and C=O/Si-O groups, respectively. Although the number of peaks was consistent with that in EVA/ATH, the intensity of the peaks changed. The addition of PhVPOSS resulted in a decrease in the intensity of the Al-O peak and an increase in the intensity of the C=O/Si-O peak. This is because the element Si in PhVPOSS reduces the proportion of Al by combining with C and O and increasing the content of the other elements. The Si spectrum in EVA/ATH/PhVPOSS was dominated by two peaks, 103.33 ev and 102.28 ev, which correspond to the Si-O and Si-C groups, respectively. The existence of the Si-C and Si-O bonds verifies that PhVPOSS is linked in the EVA molecular chains, contributing to continuous and dense char and excellent flame retardancy.

#### 3.4.2. TG-IR Analysis

**Figure 10:**
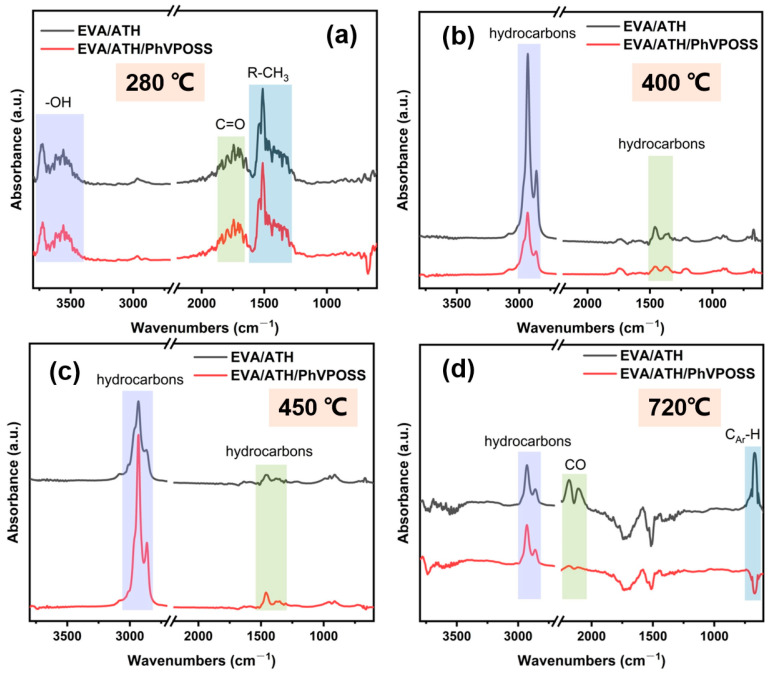
FTIR spectra of pyrolysis products of EVA/ATH and EVA/ATH/PhVPOSS at (**a**) 280 °C;, (**b**) 400 °C, (**c**) 450 °C, and (**d**) 720 °C.

Figure 10 shows the FTIR spectra of the EVA/ATH and EVA/ATH/PhVPOSS composites at specific temperatures, and the assignment of the absorption peaks is presented in Table 5. At 280 °C, EVA lost an acetic acid group, and the absorption peaks of -OH/water (3735,3558 cm^−1^), C=O (1754 cm^−1^), and R-CH_3_ (1460 cm^−1^) appeared in the spectrogram. The number and strength of the absorption peaks of EVA/ATH and EVA/ATH/PhVPOSS composites at this temperature were the same, thus indicating that PhVPOSS replaces part of ATH and has no effect on the de-acetic acid process of EVA. At 400 and 450 °C, the main chain of EVA broke and released alkanes (2930 cm^−1^), olefin (3087 and 1639 cm^−1^) small molecules, and small amounts of aromatic compounds (669 cm^−1^). The number and position of peaks were the same before and after the addition of PhVPOSS, which indicates that no new compounds appeared in the gas phase. That is, the decomposition of EVA has not changed. However, the absorption peak of EVA/ATH was stronger at 400 °C, while the absorption peak of EVA/ATH/PhVPOSS reached its maximum at 450 °C, indicating that PhVPOSS enhances the high-temperature stability of EVA and delays the degradation process of the main chain. The decomposition of the residual char occurred at 720 °C. At this temperature, EVA/ATH had more absorption peaks for CO and aromatic compounds than EVA/ATH/PhVPOSS, indicating that EVA/ATH/PhVPOSS reduces degradation and leaves more organic matter in the char residue at higher temperatures.

**Figure 11:**
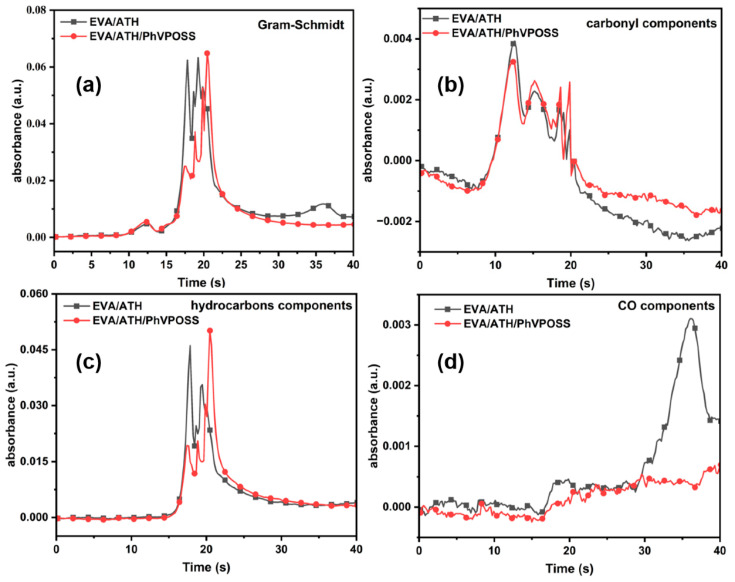
(**a**) Gram–Schmidt curves and absorption of (**b**) carbonyl compounds (1754 cm^−1^), (**c**) hydrocarbon components (2930 cm^−1^), and (**d**) CO (2180 cm^−1^) for EVA/ATH and EVA/ATH/PhVPOSS.

The Gram–Schmidt curves, carbonyl components, hydrocarbon components, and CO in the TG-FTIR spectra are shown in Figure 11. The Gram–Schmidt curves reflect the variation in the absorption intensity of the pyrolysis gas with time. Figure 11 shows that with the addition of PhVPOSS, the maximum gas released from the EVA/ATH/PhVPOSS composite was delayed, and the total amount of gas released was less. The amount of carbonyl release can reflect the process of EVA deacidification; the absorption curve of carbonyl before and after the addition of PhVPOSS was not different from the graph, which indicates that the addition of PhVPOSS does not affect the process of EVA deacidification. The hydrocarbons reflect the degradation of the EVA backbone; Figure 11c shows that PhVPOSS delays the process of EVA backbone degradation. At high temperatures, the char residue will continue to decompose. In Figure 11d, EVA/ATH continued to release CO at high temperatures, while the EVA/ATH/PhVPOSS composites showed no increase in CO release; this may be due to the addition of PhVPOSS causing the composites to form a dense vitrified char at high temperatures, thus enhancing the barrier effect of char residue.

#### 3.4.3. In-Depth Flame-Retardant Mechanisms

**Figure 12:**
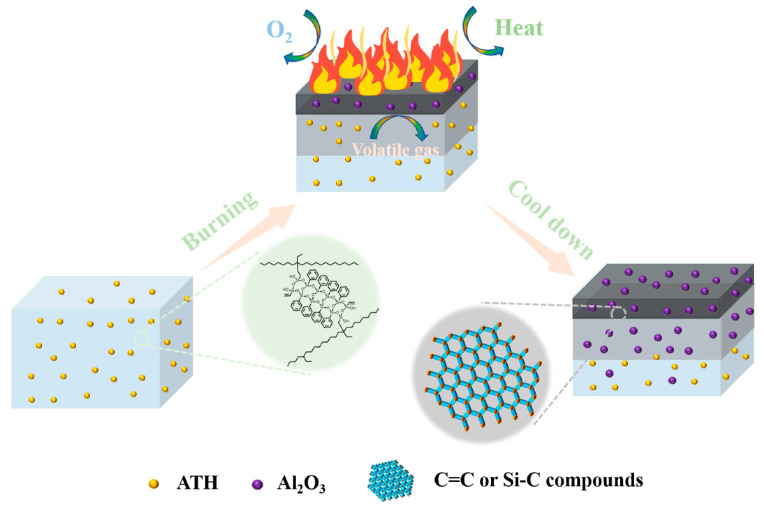
Flame-retardant mechanism for EVA/ATH/PhVPOSS composite.

Based on the above analysis of the condensed phase for the char residues and volatile pyrolysis products, we proposed a possible flame-retardancy mechanism of EVA/ATH/PhVPOSS (Figure 12). When EVA/ATH composites without PhVPOSS are burned, the residual char formed by EVA is little, and the decomposition of aluminum hydroxide produces loose alumina particles. Finally, the char with a loose structure and many holes is formed. This loose char is unable to block the transfer of heat, resulting in the continuous degradation of EVA molecular chains to produce more heat and smoke. When PhVPOSS is added to the EVA/ATH composites, PhVPOSS is grafted onto the EVA molecular chain during processing. The composite forms Si-C and Si-O bonds when it burns, making the resulting residual char more resistant to high temperatures. The char residues consisting of such structures are more continuous and denser, with no pores to suppress the exchange of oxygen, heat, and fuel, and thereby providing better protection for the inner portion of the composite.

## 4. Conclusions

EVA/ATH/PhVPOSS nanocomposites were successfully prepared by melt-blending PhVPOSS, ATH, and EVA. The reactive PhVPOSS was successfully grafted in EVA molecular chains during melting blending so that its molecular-level dispersion could be achieved during processing. By delaying thermal decomposition and increasing the residual weight, the PhVPOSS improved the thermal stability of EVA and EVA/ATH to some extent, separately. Moreover, EVA/ATH/PhVPOSS has shown better fire safety, with a shorter after-flame time; higher UL-94 rating; and lower release rate of heat, smoke, and CO. It is attributed to the formation of Si-C bonds and Si-O bonds during heating and combustion, which allows EVA/ATH/PhVPOSS composites to form a more continuous and dense char layer. It is the enhancement of charring behaviors that leads to a more effective barrier effect for the spread of decomposed gas products and heat. Therefore, reactive PhVPOSS provides new environmentally friendly candidate flame-retardant ideas for EVA/ATH composites with low smoke, good thermal stability, and flame retardancy.

## Figures and Tables

**Table 1:** Formulations of EVA/ATH/PhVPOSS composites.

Sample	Composition (wt%)		
	EVA	ATH	PhVPOSS
EVA	100	0	0
EVA/PhVPOSS	95	0	5
EVA/ATH	40	60	0
EVA/ATH/PhVPOSS	40	55	5

**Table 2:** TGA date for EVA, EVA/PhVPOSS, EVA/ATH, and EVA/ATH/PhVPOSS composites.

Sample	T_5%_ (°C)	R_max1_ (wt%/min)/T_max1_ (°C)	R_max2_ (wt%/min)/T_max2_ (°C)	Residues at 800 °C (wt%)
EVA	344.7	0.30/359.91	2.50/466.59	0.40
EVA/PhVPOSS	346.2	0.28/360.01	2.52/468.10	2.56
EVA/ATH	289.3	0.36/316.17	1.02/469.50	38.15
EVA/ATH/PhVPOSS	297.8	0.30/325.22	1.06/470.81	40.69

**Table 3:** LOI and UL-94 data for EVA, EVA/PhVPOSS, EVA/ATH, and EVA/ATH/PhVPOSS composites.

Samples	LOI (%)	UL-94 (3.2 mm)			
		Rating	t_1_ (s)	t_2_ (s)	Dripping
EVA	19.2	NR	>60	-	Yes
EVA/PhVPOSS	20.4	NR	>60	-	Yes
EVA/ATH	34.3	NR	2	>60	No
EVA/ATH/PhVPOSS	34.4	V-1	1	16	No

**Table 4:** Results of cone calorimeter tests for EVA, EVA/PhVPOSS, EVA/ATH, and EVA/ATH/PhVPOSS composites.

Sample	TTI (s)	p-HRR (kW/m^2^)	THR (MJ/m^2^)	p-SPR (m^2^/s)	TSR (m^2^/m^2^)	p-COP (g/s)	p-CO_2_P (g/s)
EVA	65	1000.56	117.31	0.1124	1538.82	0.0069	0.49
EVA/PhVPOSS	67	766.32	118.95	0.1228	1698.73	0.0053	0.39
EVA/ATH	90	180.60	76.70	0.0298	838.26	0.0022	0.09
EVA/ATH/PhVPOSS	84	166.35	71.97	0.0127	495.22	0.0002	0.09

**Table 5:** Identification of characteristic absorption peaks of gaseous decomposition products.

Wavenumber (cm^−1^)	Assignment
3735 and 3558	-OH stretching vibration or water
3087	=C-H stretching vibration
2933 and 2860	R-CH_3_ stretching vibration of acetic acid
1754	-C=O stretching vibration
1639	C=C stretching vibration
1583	Aromatic rings vibration
1213	C-O/C-O-C stretching vibration
912	=C-H deformation vibration
669	C_Ar_-H deformation vibration

## Data Availability

The data presented in this study are available upon request from the corresponding author.
